# Behavioural notes and attraction on Lepidoptera around the Gehry's Biodiversity Museum (Causeway, Calzada de Amador, Panamá, República de Panamá)

**DOI:** 10.3897/BDJ.5.e11410

**Published:** 2017-03-02

**Authors:** Patricia Esther Corro-Chang

**Affiliations:** 1 Predoctoral Student, Universidad de Panama, Programa Centroamericano de Entomologia, Panama, Panama

**Keywords:** Richness of species, abundance, butterflies, moths, Causeway.

## Introduction

Monterrubio et al. (2013) suggest ecotourism as a mechanism for environmental conservation, economical growth, and improvement of local livelihoods. The improvement of a botanical garden in Frank Gehry's new attraction, the Biodiversity Museum, promises a new source for environmental conservation of the nearby areas and as a butterfly house near the city of Panamá. The new building painted with bright colors represents the ethnical groups in Panamá, is a tribute to the wealth of fauna and flora within Panamá, and is a message to the world about how the Panamanian isthmus changed the world forever. Behind the museum there is more to discover and on the walls of this building, each day, thousand of species are interacting, resting and completing their lives histories. With this contribution we would like to: *a.)* study the diversity of Lepidoptera on the MBG, *b.)* generate a preliminary species checklist and *c.)* report notes of behavior including interactions between Lepidoptera and new habitats made by human intervention; particularly detaching the role of bright colors, and the addition of a botanical garden in the nearby areas, as possible attractants for the insects.

## Materials and Methods

**Study Area**

This study was carried out in the botanical garden and near-by areas of the Gehry's Biodiversity Museum MBG (8°55'55.5"N, 79°32'41.7"W), Causeway, Calzada de Amador, Panamá Province, República de Panamá. The botanical garden covers 3 hectares of land, five different stations that recover the ethno botanical impact of plants through natural medicine, production and agriculture by the traditional use of different grasses in the erosion control of the soils. The Causeway is compound of four small islands by the Pacific entrance to the Panama Canal which are joined by a road (the Causeway), linked by rocks extracted from the Corte Culebra during the construction of the Panama Canal in 1913. The study area is characterized as coastal, interfered aquatic habitat with patches of forest. Most of them, modified by the human activity and constructions. The annual mean temperature is 28°C, with an annual precipitation of 1642 mm and it is located at sea level.

**Sampling and Preservation**

Specimens were collected with entomological nets, killed by a soft pressure over the thorax, and stored in wax paper envelopes. All the biological material collected was stored at 0°C, in hermetic plastic boxes until it was processed in lab facilities at Programa Centroamericano de Maestría en Entomología (PCMENT), Universidad de Panamá. Each envelope contained the collection data label, including the names of the collectors and the dates. Observation and collection were done daily, and continuously during June of 2014 and March of 2005; between 08:00 to 13:00 hours near to the main entrance of the museum in a 50 m long transect that includes bushes and shrubs that attract butterflies, including *Lantana camara*, during sunny days without heavy rains or strong winds. Specimens were set with #1 and #2 entomological pins. Processing and spreading techniques for Lepidoptera samples were done following Triplehorn and Johnson (2005).

**Identification**

Determinations were made by comparisons with the material previously deposited at the Programa Centroamericano de Maestría en Entomología (Universidad de Panamá) already identified by specialists, and by review of DeVries (1987). We consulted Lamas (2004), Atlas of Neotropical Lepidoptera for updates on taxonomy and names of Papilionoidea and Hesperiioidea; Heppner (1995), Heppner (1996) for updates of Pyraloidea and Sphingoidea; Gardwood and Leeman (2011), Gardwood and Leeman (2013), Garwood and Leeman (2012) for recognition of the species during their flight; and Korytkowski (2013) for taxonomical keys to the moth groups. We also reviewed the classic literature to comprehend the descriptions of Panamanian moths collected during the Biological Survey (1910-1912) along the Panama Canal Area (Busck 1914, Dyar 1914). Part of the material collected during the research is deposited at the PCMENT entomology collection and in the private collection of the author.

## Results and Discussion

**Figure 1.**
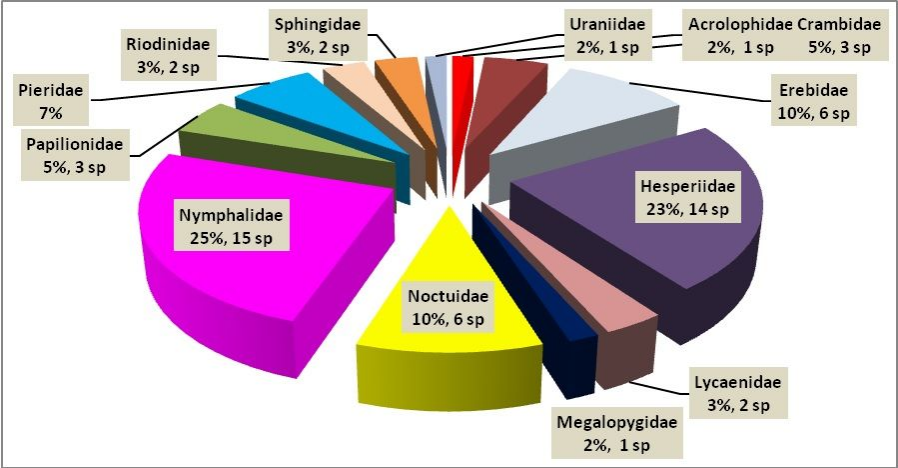
Percent representation of the abundance per Lepidoptera family.

**Figure 2.**
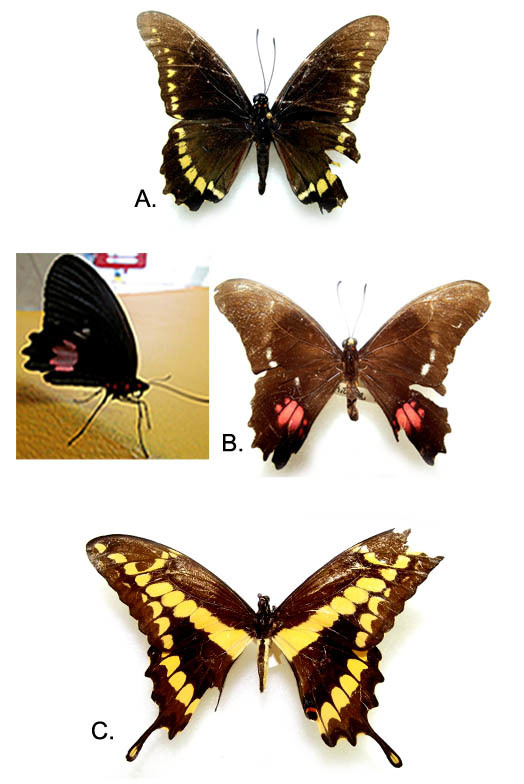
Papilionidae: A) *Battus polydamas*; B) *Parides anchisiades*; C) *Heraclides rumiko*.

**Figure 3.**
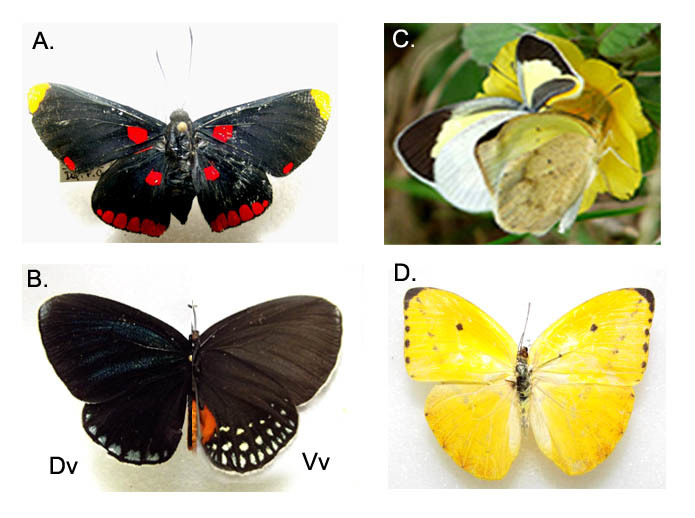
Riodinidae: A) *Melanis pixie*; B) *Eumaeus godartii Dv:dorsal; Vv:ventral.* Pieridae: C) *Eurema daira eugenia*; D) *Phoebis argante*.

**Figure 4.**
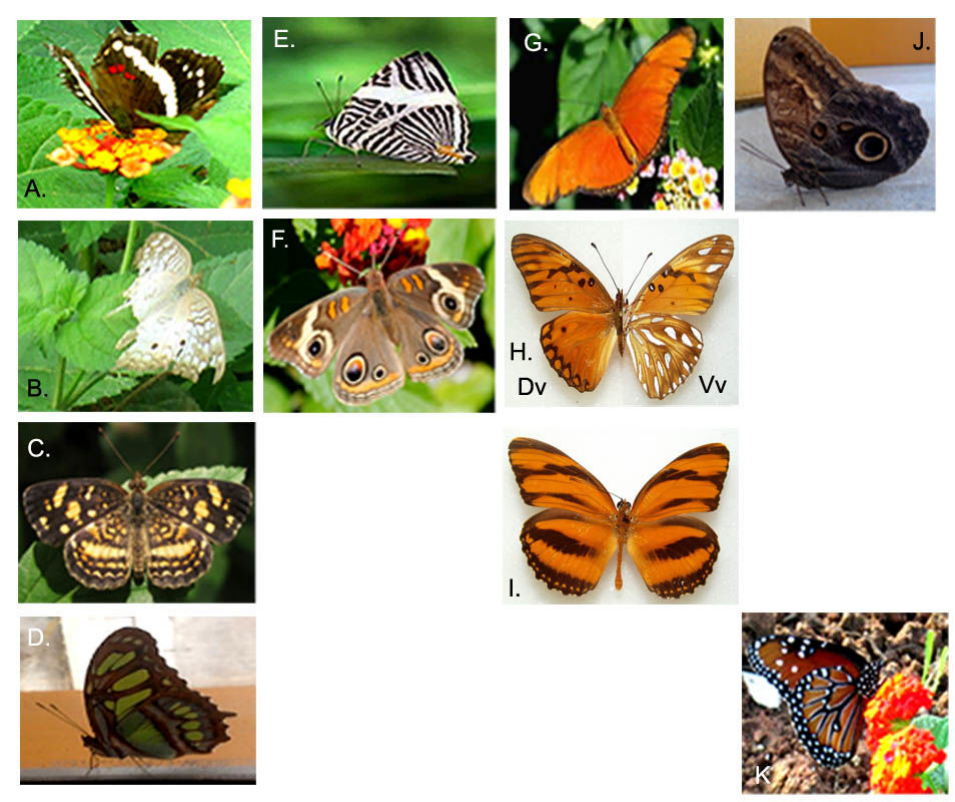
Nymphalidae. Nymphalinae: A) *Anartia fatima*; B) *A. jatrophae*; C) *Anthanassa tulcis*; D) *Siproeta stelenes*; E) *Colobura dirce*; F) *Junonia evarete.* Heliconiinae: G) *Dryas iulia*; H) *Agraulis vanilla*; I) *Dryadula phaetusa.* Morphinae: J) *Caligo atreus*; Danainae: K) *Danaus gillipus*.

**Figure 5.**
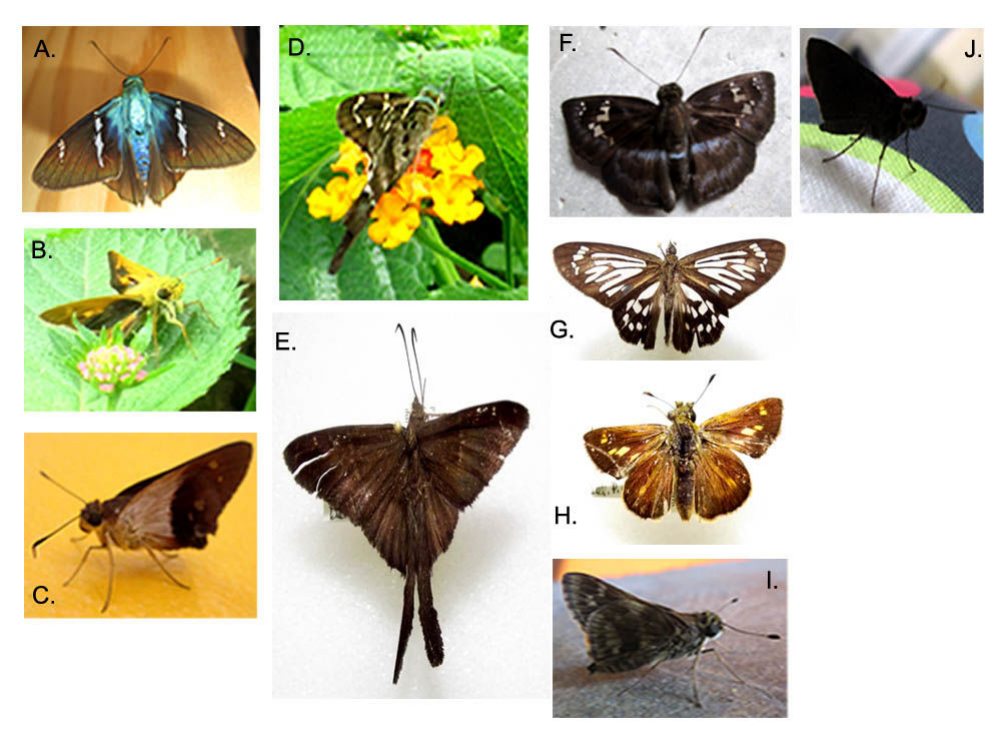
Hesperiidae: A) *Astraptes fulgerator*; B) *Anthoptus epictetus*; C) *Saliana esperi*; D) *Urbanus dorantes*; E) *U. procne*; F) *Quadrus cerialis*; G) *Phanus marshalli*; H) Parphorus nr. oeagrus; I) *Pellicia arina*; J) *Bolla* sp.

**Figure 6.**
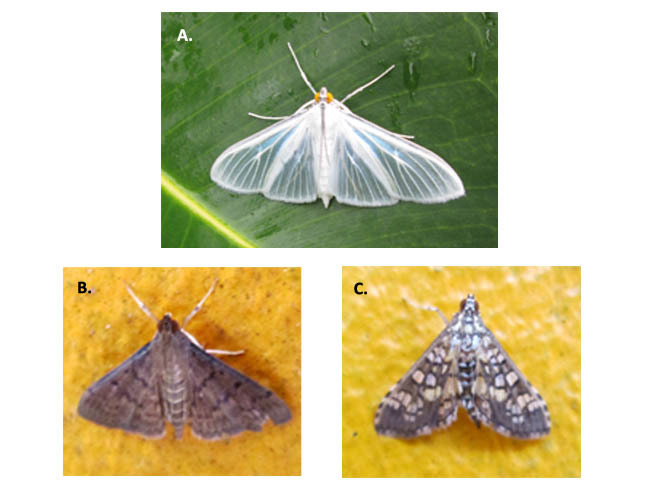
Crambidae: A) *Palpita flegia*; B) *Herpetogramma phaeopteralis*; C) *Samea ecclesialis*.

A total of 326 specimens, representing 13 families (6 butterfly and 8 moth families) distributed in 52 genera and 60 species were collected at the MBG (Table 1). The highest proportion of the abundance was found in Nymphalidae (32%, Fig. 4) and Hesperiidae (20%, Fig. 5) representing diurnally active Lepidoptera (Fig. 1); Papilionidae (5%) represented by 3 species (Fig. 2) and Pieridae (7%) with 4 species (Fig. 3). Moths were most abundant in the families Uraniidae (8%), Erebidae (Fig. 9) and Crambidae (Fig. 6), each one of them represented by a 5% of the total (Fig. 1). Richness in this work was represented by the families Nymphalidae and Hesperiidae with a total of 15 and 14 species, respectively. Meanwhile, moth richness was highly represented by 5 species corresponding to the family Erebidae (Fig. 1, Fig. 9).

The confection of the present checklist (Table 1) is a compilation of the species that inhabit the coastal ecosystem of Amador, day by day. The area is visited by tourists from all over the world that keep their sight in this attractive point of Panama. MBG has a strategic position, is surrounded by the Pacific Ocean, and which shows the contrast between the city of Panama and a non intervened main land of Fort Kobbe and Veracruz, that still non-urbanized. The distance between Fort Kobbe (largely vegetated) and the Causeway shows that most of the species could be flying actively across the narrow strip of ocean and stopping at the MBG botanical garden exploiting food sources and new habitats provided by human intervention. In addition, the bright color of the building attracts the attention of many species of birds and butterflies (Suppl. materials 1, 2). Other researchers suggests that constructions such as buildings and wind power facilities could be involved in species mortality during their daily activity; they frequently retrieve carcasses of bats, birds and insects (Kerns and Kerlinger 2004; Piorkowski 2006; Long et al. 2010). Studies in Scandinavia demonstrate that the addition of bright color and different constructions, such as wind turbines, plays an important role on the fauna with impacts on their activities including their migratory patterns (Ahlén et al. 2007), if we compare the results from this study and the preliminary observations of our work it is possible to create relationships with the Gehry's museum situation. It is contributing to insect activities, is an attractant, and also is an artificial barrier during bird migratory season (Fig. 8). Studies on the impacts including effects of new architectural concepts still leave gaps in our knowledge (Long et al. 2010) and particularly in Panama; this is the first contribution in this topic.

**Figure 7.**
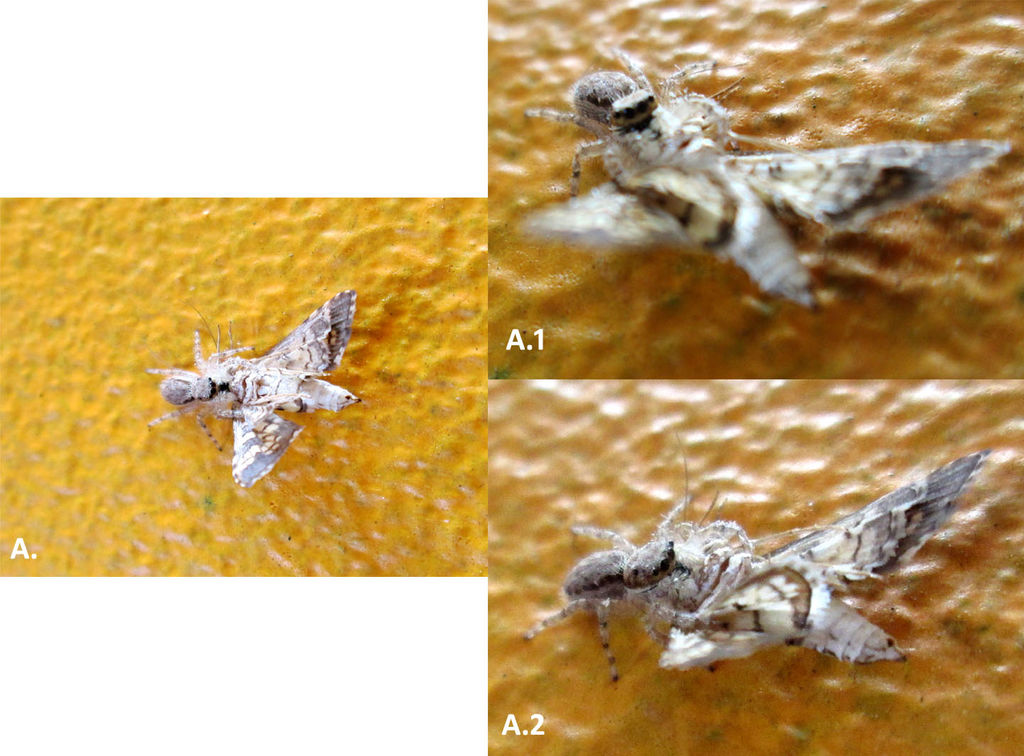
A female of *Pelegrina variegata* Pickard-Cambridge F., 1901 predating *Samea ecclesialis* Guenée, 1854 perched on the walls of the Gehry's Biodiversity Museum.

**Figure 8.**
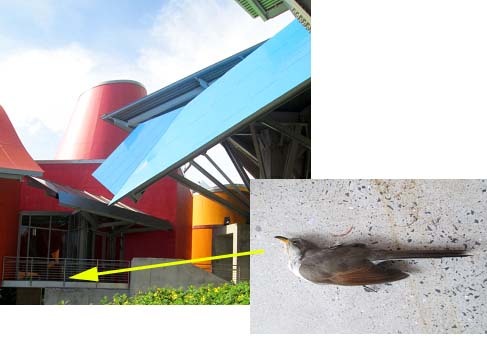
Some birds hit the glass of the Gehry's temporary exhibition corner during their migratory season.

**Figure 9.**
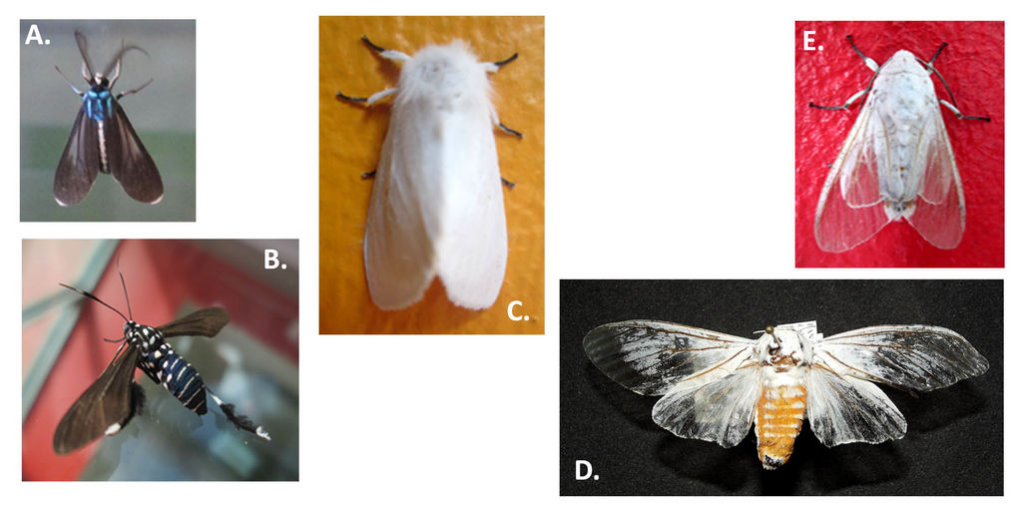
Erebidae: A) *Uranophora leucotelus*; B) *Horama plumipes*; C) *Spilosoma congrua*; D) *Halysidota* sp.; E) *Munona iridescens*.

Some notes related to behavior and interaction patterns were registered over the colored surfaces of the building (Suppl. materials 1, 2). *Saliana esperii* shows a particular preference for the yellowish surfaces of the building; many times, this species was observed visiting the walls and posing over it, extending its proboscis and constantly searching for nutritional sources on different portions of the wall surface. Walls of the MBG building also function as an arena for predators, developing complex nets of interactions (Fig. 7). Females of the species *Pelegrina variegata* Arachnida: Salticidae, are frequent all over the walls of the building, opportunistically catching various species of insects that lie on different portions of the wall. As of this work we have witnessed predation of the crambid moth *Samea ecclesialis* by *P. variegata* and its preference for this particular species of moth (Fig. 6, Fig. 7). The yellow walls of the building also serve as resting place for *Urania fulgens* during their migration across Panama in the months of March and June, each year (Smith 1983). But glass of the building also represents a risk to other species such as birds during their migration times, many species of bird hit the window glass (Fig. 8). Meanwhile, diurnal moths such as *Horama plumipes*, lie on the glass that connects to the Gehry's temporary exhibits, displaying their perfectly mimicry to wasps of the family Sphecidae (Fig. 9).

## Conclusion

The coastal ecosystem of Amador has a rich Lepidoptera fauna, besides the strong human intervention in this area; species exploit the various sources that the MBG offers. The addition of colors, a botanical garden and shapes on this construction serve as attractants for day active species and variety of interactions. This work represents a preliminary contribution to our knowledge of the species of Lepidoptera active in the Calzada de Amador, and will answer many of the questions asked by visitors interested in the fauna of this important tourist point of Panamá, thus promoting its conservation.

## Supplementary Material

Supplementary material 1 Biodiversity museum before the opening, October of 2014. Data type: Image
File: oo_111042.jpg Patricia Esther Corro Chang

Supplementary material 2 Lepidoptera attracted by colors of the Gehry's building Data type: Images
File: oo_111051.jpg Patricia Esther Corro Chang

## Figures and Tables

**Table 1.** Checklist of Lepidoptera species around the Gehry's Biodiversity Museum

**Family**	**Subfamily**	**Tribe**	**Species**	**Common name** (English)*
Acrolophidae			*Acrolophus panamae* Busck, 1914	Panama Grass Tube-worm Moth
Crambidae	Pyraustinae		*Herpetogramma phaeopteralis* (Guenée, 1854)	Sod Webworm Moth
Crambidae	Pyraustinae	Spilomelini	*Palpita flegia* (Cramer, 1777)	
Crambidae	Pyraustinae	Spilomelini	*Samea ecclesialis* Guenée, 1854	
Erebidae	Arctiinae		*Calonotos metallicus* Druce, 1884	
Erebidae	Arctiinae	Arctiini	*Halysidota* sp.	
Erebidae	Arctiinae	Arctiini	*Horama plumipes* (Drury, 1773)	Wasp moth
Erebidae	Arctiinae	Arctiini	*Munona iridescens* Schaus, 1894	
Erebidae			*Spilosoma congrua* (Walker, 1855)	Agreeable Tiger Moth
Erebidae			*Uranophora leucotelus* (Butler, 1876)	
Hesperiidae	Hesperiinae	Anthoptini	*Anthoptus epictetus* (Fabricius, 1793)	Trailside Skipper
Hesperiidae	Pyrginae	Eudamini	*Astraptes fulgerator* (Walch, 1775)	Two-barred Flasher
Hesperiidae	Pyrginae	Eudamini	*Astraptes talus* (Cramer, 1777)	Green Flasher
Hesperiidae	Pyrginae	Pyrgini	*Bolla cupreiceps* (Mabille, 1891)	Copper-headed Sootywing
Hesperiidae	Hesperiinae	Calpodini	*Carystoides lebbaeus* (Hewitson, 1876)	Lebbaeus Rubyeye
Hesperiidae	Pyrginae	Pyrgini	*Nisoniades panama* Evans, 1953	Panamanian Tufted- Skipper
Hesperiidae	Hesperiinae	Moncini	*Parphorus nr. oeagrus* (Godman, 1900)	Tawny-washed Skipper
Hesperiidae	Pyrginae	Pyrgini	*Pellicia arina* Evans, 1953	Glazed Tufted- Skipper
Hesperiidae	Pyrginae	Eudamini	*Phanus marshalli* (W.F. Kirby, 1880)	Common Ghost Skipper
Hesperiidae	Pyrginae	Achlyodini	*Quadrus cerialis* (Stoll, 1782)	Common Blue- Skipper
Hesperiidae	Hesperiinae	Calpodini	*Saliana esperi* Evans, 1955	Saliana
Hesperiidae	Pyrginae	Pyrgini	*Staphylus ascalaphus* (Staudinger, 1876)	Central American Sootywing
Hesperiidae	Pyrginae	Eudamini	*Urbanus dorantes* (Stoll, 1790)	Dorantes Longtail
Hesperiidae	Pyrginae	Eudamini	*Urbanus procne* (Plötz, 1880)	Brown Longtail
Lycaenidae	Theclinae	Eumaeini	*Calycopis drusilla* Field, 1967	Drusilla Groundstreak
Lycaenidae	Theclinae	Eumaeini	*Magnastigma hirsuta* (Prittwitz, 1865)	Hirsuta Hairstreak
Megalopygidae	Megalopyginae		*Megalopyge lanata* (Stoll, 1780)	Mangrove Flannel Moth
Noctuidae	Catocalinae		*Anticarsia gemmatalis* (Hübner, 1818)	Velvetbean Caterpillar Moth
Noctuidae	Catocalinae		*Ascalapha odorata* Linnaeus, 1758	Black Witch
Erebidae			*Letis* sp.	Marbled Witch
Noctuidae	Plusiinae		Noctuid sp. 1	
Erebidae	Catocalinae		Noctuid sp. 2	
Noctuidae	Amphipyrinae		*Spodoptera* sp.	
Nymphalidae	Heliconiinae	Heliconiini	*Agraulis vanillae* (Linnaeus, 1758)	Passion butterfly
Nymphalidae	Nymphalinae	Kallimini	*Anartia fatima* (Fabricius, 1793)	Banded Peacok
Nymphalidae	Nymphalinae	Kallimini	*Anartia jatrophae* (Linnaeus, 1763)	White Peacok
Nymphalidae	Nymphalinae	Phyciodini	*Anthanassa frissia tulcis* (H.W.Bates, 1864)	Pale-banded Crescent
Nymphalidae	Brassolinae	Brassolini	*Brassolis isthmia* Bates, 1864	Small-spoted Owlet
Nymphalidae	Morphinae	Brassolini	*Caligo atreus* (Kollar, 1850)	Atreus/ Banded Giant Owl
Nymphalidae	Nymphalinae	Coeini	*Colobura dirce* (Linnaeus, 1758)	Dirce/Small Beauty
Nymphalidae	Danainae	Danaini	*Danaus gillipus thersippus* (H.W.Bates, 1863)	Queen
Nymphalidae	Heliconiinae	Heliconiini	*Dryadula phaetusa* (Linnaeus, 1758)	Banded Orange
Nymphalidae	Heliconiinae	Heliconiini	*Dryas iulia* (Fabricius, 1775)	Julia
Nymphalidae	Heliconiinae	Argynnini	*Euptoieta hegesia* (Cramer, 1779)	Mexican Fritillary
Nymphalidae	Biblidinae	Biblidini	*Hamadryas laudamia* (Cramer, 1777)	Starry Night
Nymphalidae	Nymphalinae	Kallimini	*Junonia evarete* (Cramer, 1779)	Tropical Buckeye
Nymphalidae	Brassolinae	Brassolini	*Opsiphanes cassina* C. Felder & R. Felder, 1862	Split-banded Owlet
Nymphalidae	Nymphalinae	Kallimini	*Siproeta stelenes* (Linnaeus, 1758)	Malachite
Papilionidae	Papilioninae	Troidini	*Battus polydamas* (Linnaeus, 1758)	Polydamas Swallowtail
Papilionidae	Papilioninae	Papilionini	*Heraclides rumiko* (Shiraiwa & Grishin, 2014)	Western-giant Swallowtail
Papilionidae	Papilioninae	Troidini	*Parides anchisiades farfan* K.S. Brown, 1994	Anchisiades Cattleheart
Pieridae	Coliadinae	Coliadini	*Eurema arbela gratiosa* (Doubleday, 1847)	Disjunct Yellow
Pieridae	Coliadinae	Coliadini	*Eurema daira eugenia* (Wallengren, 1860)	Barred Yellow/ Barred sulphur
Pieridae	Coliadinae	Coliadini	*Phoebis argante* (Fabricius, 1775)	Apricot Sulphur
Pieridae	Coliadinae	Coliadini	*Phoebis sennae* (Linnaeus, 1758)	Cloudless Sulphur
Riodinidae	Theclinae	Eumaeini	*Eumaeus godartii* (Boisduval, 1870)	White-tipped Cycadian
Riodinidae	Riodininae	Riodinini	*Melanis pixe sanguinea* (Stichel, 1910)	Red-bordered Pixie
Sphingidae	Macroglossinae	Dilophonotini	*Isognathus scyron* (Cramer, 1780)	
Sphingidae	Sphinginae	Sphingini	*Manduca rustica rustica* (Fabricius, 1775)	Rustic Sphinx
Uraniidae	Uraniinae		*Urania fulgens* (Walker, 1854)	Urania Swallowtail
