# Supplementary figures and images for: Behavioural notes and attraction on Lepidoptera around the Gehry's Biodiversity Museum (Causeway, Calzada de Amador, Panamá, República de Panamá)
Source: Biodivers Data J. 2017 Mar 2;(5):e11410. doi: 10.3897/BDJ.5.e11410 (PMC5345114; doi:10.3897/BDJ.5.e11410)

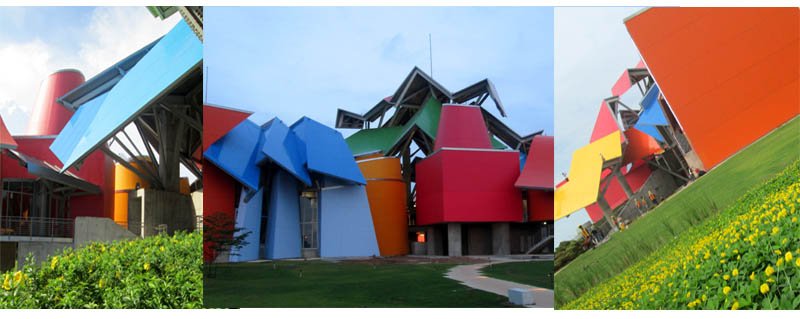

Supplement: Supplementary material 1 — Biodiversity museum before the opening, October of 2014. [file bdj-05-e11410-s001.jpg]

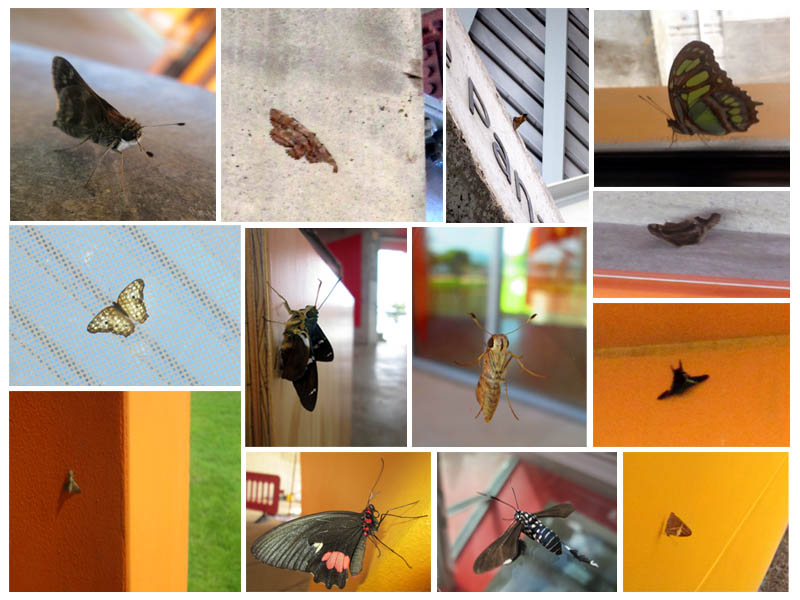

Supplement: Supplementary material 2 — Lepidoptera attracted by colors of the Gehry's building [file bdj-05-e11410-s002.jpg]
